# Effect of Acute, Slightly Increased Intra-Abdominal Pressure on Intestinal Permeability and Oxidative Stress in a Rat Model

**DOI:** 10.1371/journal.pone.0109350

**Published:** 2014-10-08

**Authors:** Yuxin Leng, Kuo Zhang, Jie Fan, Min Yi, Qinggang Ge, Li Chen, Lu Zhang, Gaiqi Yao

**Affiliations:** 1 Department of Intensive Care Unit, Peking University Third Hospital, Haidian District, Beijing, P.R. China; 2 Laboratory Animal Center, Peking University Health Science Center, Haidian District, Beijing, P.R. China; 3 Department of Central Laboratory, Peking University Third Hospital, Haidian District, Beijing, P.R. China; 4 Department of Gastroenterology, Peking University Third Hospital, Haidian District, Beijing, P.R. China; INSERM, France

## Abstract

**Introduction:**

Intra-abdominal hypertension (IAH) is known as a common, serious complication in critically ill patients. Bacterial translocation and permeability changes are considered the pathophysiological bases for IAH-induced enterogenic endotoxemia and subsequent multiorgan failure. Nevertheless, the effects of slightly elevated intra-abdominal pressures (IAPs) on the intestinal mucosa and the associated mechanisms remain unclear.

**Methods:**

To investigate the acute effects of different nitrogen pneumoperitoneum grades on colonic mucosa, male Sprague-Dawley rats were assigned to six groups with different IAPs (0 [control], 4, 8, 12, 16, and 20 mmHg, n = 6/group). During 90 min of exposure, we dynamically monitored the heart rate and noninvasive hemodynamic parameters. After gradual decompression, arterial blood gas analyses were conducted. Thereafter, structural injuries to the colonic mucosa were identified using light microscopy. Colon permeability was determined using the expression of tight junction proteins, combined with fluorescein isothiocyanate dextran (FD-4) absorption. The pro-oxidant-antioxidant balance was determined based on the levels of malondialdehyde (MDA) and antioxidant enzymes.

**Results:**

IAH significantly affected the histological scores of the colonic mucosa, tight junction protein expression, mucosal permeability, and pro-oxidant-antioxidant balance. Interestingly, elevations of IAP that were lower than the threshold for IAH also showed a similar, undesirable effect. In the 8 mmHg group, mild hyponatremia, hypocalcemia, and hypoxemia occurred, accompanied by reduced blood and abdominal perfusion pressures. Mild microscopic inflammatory infiltration and increased MDA levels were also detected. Moreover, an 8-mm Hg IAP markedly inhibited the expression of tight junction proteins, although no significant differences in FD-4 permeability were observed between the 0- and 8-mmHg groups.

**Conclusions:**

Acute exposure to slightly elevated IAP may result in adverse effects on intestinal permeability and the pro-oxidant-antioxidant balance. Therefore, in patients with critical illnesses, IAP should be dynamically monitored and corrected, as soon as possible, to prevent intestinal mucosal injury and subsequent gut-derived sepsis.

## Introduction

Intra-abdominal hypertension (IAH, sustained elevation of intra-abdominal pressure above 12 mmHg in adults and above 10 mmHg in children)is currently known as a common, serious complication in critically ill patients [1]. According to the 2007 consensus of the World Society of the Abdominal Compartment Syndrome (WSACS), the incidence of IAH ranges from 30% to 70% [2], with an incidence of 30–40% in intensive care unit patients [3]. Without timely and appropriate intervention, IAH may result in abdominal compartment syndrome (ACS), which is closely related to the pathophysiological changes caused by deteriorations in organ perfusion and microcirculation [4].

Among the organ systems frequently affected by IAH, the intestine is the most susceptible. Ischemic injury and subsequent reperfusion-induced oxidative damage are involved in the development of IAH [5]–[7].Intestinal ischemia triggers the enhancement of intestinal permeability, bacterial translocation, and the subsequent systematic inflammatory response. These, in turn, cause capillary leakage that leads to bowel edema, thus further worsening the IAH and leading to a morbidity-ischemia cycle [8]–[11]. Recently, Cheng et al. directly detected the pathophysiological basis of IAH-induced intestinal damage. They observed that rabbit intestinal microcirculatory blood flow was reduced by 40% after 2 h of 15-mmHg IAP. The blood flow was further reduced to 81% when the IAP was increased to 25 mmHg for 6 h [12]. The IAH-induced endotoxemia, following increased permeability, may be correlated with tight junction (TJ) damage.

Although the adverse effects of IAH have received much attention, the effects of slightly elevated IAPs remain unclear. Despite critically ill patients, the intraperitoneal pressures in adults are rarely more than 5–7 mmHg [13]–[16], which is within the acceptable normal range for such patients[1], [17]. In fact, based on the IAH diagnostic standards (12 mmHg for adults and 10 mmHg for children), 60% of adults in the intensive care unit have slightly elevated IAPs of 5–12 mmHg; the corresponding range for children is approximately 4–10 mmHg [1]. This level of IAP is also considered to be dangerous. Sfez et al. and Baroncini et al. observed that mild elevations of IAP (6–12 mmHg), induced by the carbon dioxide pneumoperitoneum that is commonly used during laparoscopic surgeries, influences the respiratory and cardiocirculatory parameters of children [18], [19]. Mogilneret al. demonstrated that IAPs of 3 and 6 mmHg led to decreased portal vein and superior mesenteric artery flow, respectively, in rats [20].Even after considering the size of the models, 6 mmHg is still lower than the IAP threshold for IAH in similar conditions among children, which has been recently defined as 10 mmHg by the WSACS experts [1]. Thus, it is quite important to elucidate whether the physiological changes that occur under slightly increased IAPs lead to pathophysiological damage of the intestinal mucosa, the mechanisms involved in the TJ permeability and integrity changes, and whether oxidative mechanisms are involved.

Therefore, in the present study, we aimed to investigate the effects of different grades of nitrogen pneumoperitoneum (0, 4, 8, 12, 16, and 20 mmHg) on intestinal permeability and oxidative damage. We specifically hypothesized that short-term, low-grade IAP elevation, may cause adverse effects on the intestine, without any visible pathological changes.

## Materials and Methods

### Animals and Ethics Statement

Seventy-two male Sprague-Dawley rats (8-weeks-old, weighing 211.6±7.6 g)purchased from Weitonglihua Company (Beijing, China) were used for all the experiments. They were individually housed in polypropylene cages and kept under controlled environmental conditions, with 12-h light/dark cycles. The rats were allowed food and water *ad libitum*. All surgeries were conducted under sodium pentobarbital anesthesia (intraperitoneal injection, 40 mg/kg), and all efforts were made to minimize pain. After the procedures, the animals were euthanized with an overdose of sodium pentobarbital (intraperitoneal injection, 160 mg/kg) which also included2% lidocaine to minimizepain [21], [22]. The experimental protocol was approved by the Peking University Biomedical Ethics Committee-Experimental Animal Ethics Branch (Approval No. LA2013-12).

### Experiment Protocols

To study the acute effects of different grades of nitrogen pneumoperitoneum on intestinal permeability and oxidative damage, rats were randomly allocated, according to a random-number table, into six groups, corresponding to different IAPs(0 [control], 4, 8, 12, 16, and 20 mmHg; n = 6 per group).During the 90-min exposure to nitrogen pneumoperitoneum, the heart rate(HR) and noninvasive blood pressure (BP) were monitored dynamically using a BP-98A BP system (Softron, Tokyo, Japan). At 90 min, after gradual decompression, arterial blood gas (ABG) analyses were conducted, and colon tissues were obtained. We determined the antioxidant and oxidant enzymes that reflect oxidative changes. The histological damage and expression of typical transmembrane proteins (claudin 5 and occludin), responsible for TJ permeability, were assessed. Moreover, another group of rats (n = 6/group),which were subjected to the same IAP conditions, were used to determine intestinal permeability to fluorescein isothiocyanate (FITC) dextran.

### Nitrogen Pneumoperitoneum Procedure

Before the commencement of the experiments, the rats were deprived of food for 12 h, but had free access to water. After inducing anesthesia with an intraperitoneal injection of sodium pentobarbital (40 mg/kg), the rats were placed, supine in the restraining apparatus, on a heated operating table to help maintain their body temperature at 37°C during the 90-min procedure. Nitrogen pneumoperitoneum was established by the injection of nitrogen, using a disposable venous infusion needle connected to a microinfusion pump. The microinfusion pump was also linked to the BP meter to dynamically monitor the IAP. After the target IAP was achieved, a low flow of nitrogen (0–1 mL/h) was used to maintain the desired IAP.

### Monitoring Physiological Changes under Nitrogen Pneumoperitoneum

#### Dynamic Monitoring of Noninvasive BP and HR

The indirect tail cuff method was used to monitor the HR, systolic BP (SBP), and diastolic BP (DBP), as described in previous studies [23], [24].When the target IAP was achieved, the inflatable cuff was placed around the base of the tail and a piezoelectric pulse detector was positioned distal to the cuff. After 5 min of calibration, the cuff was inflated to approximately 180 mmHg. As the pressure in the cuff was released, the SBP, DBP, and HR were recorded using a physiological polygraph. Within the 90-min experiment, all parameters were recorded at 15-min intervals. The mean values were recorded as the final results.

#### Blood Gas Analysis

To determine whether slightly increased IAPs cause physiologically adverse effects, such as hypoxia, hypercapnia, acidosis, or electrolyte imbalance, 1 mL of arterial blood was immediately drawn from the abdominal aortic, through a laparotomy incision, upon discontinuation of the nitrogen pneumoperitoneum procedure. ABG analyses were performed using a blood gas analyzer(GEM premier3000, Instrumentation Laboratory, Bedford, MA, USA).

### Preparation of Colon Tissue and Detection of Histological Damage

#### Preparation of Colon Tissue

To prevent postmortem damage, the colon tissue was collected, immediately after euthanasia, gently flushed using cold saline, and divided into three sections. The whole process, including the drawing of arterial blood and sample collection, was completed within 1 min. One of the colon specimens (1 cm×1 cm) was fixed in4% paraformaldehyde for 12 h. Thereafter, 5-µmthick, paraffin-embedded sections were prepared for conventional hematoxylin-eosin (H&E) staining and immunohistochemical staining. The other two specimens were immediately frozen at −70°C for subsequent analysis of oxidative changes and Western blotting.

#### Detection of Histological Damage

Conventional H&E staining was performed to visualize histological changes that occurred during exposure to different IAPs. After staining, micrographs were obtained (Nikon E600, Nikon, Tokyo, Japan) to determine a histological score on a 0–9 scale; as shown in Table 1, with references to the works of Park et al. and Hartmann et al., the degree of inflammatory infiltration, mucosal edema, and epithelial integrity damage were evaluated [25], [26]. The scoring was performed by three observers who were blinded to the allocation. In each slide, five different fields (at ×200 magnification)were selected and analyzed; the mean value of the measurements by the three observers was recorded as the final result.

**Table 1:** Scoring criteria for histologic damage in colon sections.

Histological change	Scale (0–3)
***Inflammatory infiltration***	0, rare inflammatory cells in the lamina propria were counted [0–10/high-powered field];
	1, increased numbers of inflammatory cells in the lamina propria [11–20/high-powered field];
	2, confluence of inflammatory cells, extending to the submucosa;
	3, transmural extension of the infiltrate
***Epithelial integrity***	0, intact epithelium;
	1, a partially broken epithelial line;
	2, separated from the mucosa;
	3, epithelium completely removed or with exposed and digested lamina propria
***Mucosal edema***	0, normal;
	1, slight;
	2, intermediate;
	3, intense

#### Changes in the Pro-Oxidant-Antioxidant Balance

The levels of malondialdehyde (MDA), glutathione peroxidase (GSH-Px), catalase (CAT) and serum super oxide dismutase (SOD) in the colon were measured to determine the oxidation changes induced following exposure to elevated IAPs. After slow rewarming, the cooled samples were weighed, homogenized in saline (1∶10 w/v), and centrifuged at 3000 rpm for 10 min. The recovered supernatants were used for the following assay, according to previously described methods [27]–[30].The numerical spectrophotometric (Thermo Fisher Scientific, Waltham, MA, USA) readings were recorded. All commercial kits were provided by Nanjing Jiancheng Bioengineering Institute (Nanjing, China).

### Intestinal Permeability toFD-4 Macromolecules

The intestinal permeability to FITC-dextran (FD-4; molecular weight 4000 Da; Sigma-Aldrich, St. Louis, MO, USA, Lot. 68059.) was assessed using the method of Martin et al.[31].Briefly, after anesthetizing an animal with an intraperitoneal injection of sodium pentobarbital (40 mg/kg), a 2-cm,median laparotomy incision was made, and a 10-cm segment of the distal ileum, with preserved superior mesenteric vessels, was dissected 3 cm proximal to the cecum. The two ends of the isolated intestinal segment were ligated. One milliliter of phosphate-buffered saline (0.1 mol/L, pH 7.2) containing 25 mg of FD-4 was injected into the lumen, and the 10-cm intestinal segment was carefully returned to the abdomen, covered, and protected with gauze soaked in warm saline. After 30 min, portal venous blood samples were taken and centrifuged at 3000 ×*g* for 10 min at 4°C. The plasma and FD-4 standards were analyzed to determine the FD-4 concentrations spectrophotometrically, with an excitation wavelength of 492 nm and an emission wavelength of 518 nm.

### Immunohistochemical Staining and Western Blotting

#### Immunohistochemical Staining

Immunohistochemical staining of transmembrane proteins was performed as previously described [31]. Primary antibodies of rabbit anti-claudin-5 (1∶200; Millipore, Billerica, MA, USA; Lot. 2211525)and mouse anti-occludin (1∶400; Invitrogen, Carlsbad, CA, USA; Lot. 1204426A) were incubated overnight at 4°C, washed, and analyzed using goat anti-rabbit (PV-6001, Zhongshan Golden Bridge, Beijing, China) and anti-mouse (PV-6002, Zhongshan Golden Bridge) detection kits, respectively. Digital images were obtained using an E600microscope (Nikon).

#### Western Blotting

Western blotting analyses of transmembrane proteins were conducted according to the method of Li et al. [32]. Briefly, colon tissue samples were homogenized in lysis buffer (20 mmol/LTris-HCl [pH 7.5], 1% Triton X100, 0.2 mol/LNaCl, 2 mmol/Lethylenediamine tetraacetic acid, 2 mmol/Lethylene glycol tetraacetic acid, 1 mol/Ldithiothreitoland 2 mol/Laprotinin). Proteins (60 µg) were electrophoresed using sodium dodecyl sulfate polyacrylamidegel electrophoresis (8%) and transferred to a polyvinylidene fluoride membrane. The membranes were blocked with nonfat dried milk in tris-buffered saline containing 0.05%Tween-20 (TTBS) for 1 h at room temperature. They were then incubated—with gentle shaking, overnight, at 4°C—with the antibodies used in the immunohistochemical analysis (claudin 5: 1∶1000; occludin: 1∶500).Thereafter, goat anti-rabbit and goat anti-mouse fluorescently labeled secondary antibodies(1∶10,000; LI-COR Biosciences, Lincoln, NE, USA)were added to the membranes at room temperature for 1 h. The bound proteins were visualized following scanning of the membranes with an Odyssey Infrared Imaging System (LI-COR Biosciences).

### Statistical Analysis

Statistical analyses were performed using SPSS 16.0software (SPSS, Chicago, IL, USA).Data were analyzed for normal distribution using the Kolmogorov-Smirnov tests. As the data were normally distributed, they are expressed as means ± SD values. One-way analysis of variance was performed, followed by the Scheffe post hoc test [33]. P-values <0.05 were considered statistically significant.

## Results

### Physiological Changes at Different IAPs

#### HR and Noninvasive BP

**Figure 1:**
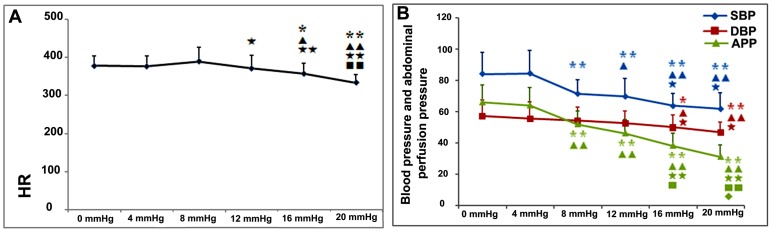
Effects of different grades of nitrogen pneumoperitoneum on heart rate (HR) and noninvasive hemodynamic parameters. A: HR; 90-min exposure to intra-abdominal hypertension caused a significant reduction in HR. Compared with the 0-mmHg group, the HR in the 12-, 16-, and 20-mmHg groups were significantly lower. B: Noninvasive hemodynamic parameters; the 90-min exposure to intra-abdominal pressures (IAPs) ≥8 mmHg caused significant reductions in the systolic blood pressure (SBP) and abdominal perfusion pressure (APP). The SBP and APP in the 8-, 12-, 16-, and 20-mmHg groups were significantly lower than those in the 0-mmHg group. As the IAPs increased, the noninvasive hemodynamic parameters deteriorated. All data are shown as means ± SD *P<0.05, **P<0.01, vs. 0 mmHg; ^▴^P<0.05, ^▴▴^P<0.01, vs. 4 mmHg; ^★^P<0.05, ^★★^P<0.01, vs. 8 mmHg; ^▪^P<0.05, ^▪▪^P<0.01, vs. 12 mmHg.

The effects of the different IAPs on the HR and noninvasive BP measurements are shown in Figure 1. The results indicated that IAPs equal to or higher than the threshold for IAH (12 mmHg in adults and 10 mmHg in children) significantly inhibited HR and BP. The abdominal perfusion pressure (APP), which can be determined by subtracting the IAP from the mean arterial pressure (MAP), was also affected and had decreased; the APP is closely related to the functioning of abdominal organs. Moreover, slight increases in IAP, to levels lower than the threshold for diagnosing IAH, also showed adverse effects on organ perfusion. An IAP of 4 mmHg did not have a significant effect on noninvasive BP parameters. However, as the IAP was increased to 8 mmHg, obvious decreases in SBP (8 vs. 0 mmHg: 71.46±9.19 vs. 84.42±13.58 mmHg; *P*<0.01) and APP (8 vs. 0 mmHg: 52.21±8.40 vs. 66.13±11.05 mmHg; *P*<0.01) were detected. These observations suggested that 8 mmHg, but not necessarily 10 or 12 mmHg, is the main causative factor for the deterioration of noninvasive hemodynamic parameters, in rats.

#### Arterial Blood Gas

Higher IAP increases (≥12 mmHg) resulted in serious adverse effects on ABG findings. Acidosis, hypoxia, hypercapnia, and electrolyte disturbances were prominent at these elevated pressures. In the 20-mmHg group, the acidosis was so severe that the pH values of all the six rats were lower than the analyzer's minimum detectable value (pH 6.8–7.8). Unexpectedly, slightly increased IAPs of 4 and 8 mmHg (lower than the threshold for IAH) also led to mild hypoxia, hypocalcemia, and hyponatremia (Table 2).

**Table 2:** Effects of the different grades of nitrogen pneumoperitoneum on arterial blood gas parameters.

Parameters (range)	0 mmHg	4 mmHg	8 mmHg	12 mmHg	16 mmHg	20 mmHg
pH (6.80–7.80)	7.35±0.37	7.32±0.23	7.32±0.33	7.25±0.05*	6.90±0.11* ^▴★^	<6.8
PO_2_ (5–115 mmHg)	77.50±12.45	74.50±13.84	61.50±7.67* ^▴^	26.17±7.20**^▴▴★★^	22.75±6.34**^▴▴★★^	20.33±8.83
PCO_2_ (0–760 mmHg)	42.50±5.17	41.00±3.58	49.00±9.86	59.83±7.78* ^▴▴^	101±25.34**^▴▴★★▪▪^	123±38.124**^▴▴★★▪▪^
SO_2_C (0–100%)	93.17%±4.07%	90.00%±3.16%	90.71%±4.80%	32.75%±11.81%**^▴▴★★^	18.25%±6.13%**^▴▴★★^	16.08%±7.02%**^▴▴★★^
Lac (0.3–15 mmol/L)	2.47±1.21	2.08±0.82	2.15±0.59	4.72±1.42**^▴▴★★^	7.28±0.70**^▴▴★★▪▪^	10.21±1.55**^▴▴★★▪▪^
Glu (1.11–27.78 mmol/L)	6.92±0.84	9.18±1.58	10.08±2.36	12.48±0.64**^▴^	16.15±6.10**^▴▴^	16.57±7.08**^▴▴^
Na (100–200 mmol/L)	135.17±3.25	128.50±2.43**	128.83±3.06**	128.50±2.88**	124.75±5.06**	120.43±6.36**
K (1–20 mmol/L)	4.80±0.52	5.05±0.64	5.30±0.83	5.10±0.40	6.80±0.96**^▴▴★★▪▪^	7.30±0.87**^▴▴★★▪▪^
Ca (0.1–5 mmol/L)	1.07±0.36	0.71±0.24**	0.73±0.10**	0.72±0.25**	0.68±0.29**	0.47±0.17**

PO_2_,partial pressure of arterial oxygen; PCO_2_, arterial carbon dioxide; SO_2_C, arterial oxygen saturation; Lac, lactic acid; Glu, glucose; Na, sodium; K, potassium; Ca,calcium.

All data are shown as means ± SD values.

*P<0.05, **P<0.01, vs. 0 mmHg; ^▴^P<0.05, ^▴▴^P<0.01, vs. 4 mmHg; ^★^P<0.05, ^★★^P<0.01 vs. 8 mmHg; ^▪^P<0.05, ^▪▪^P<0.01, vs. 12 mmHg.

### Oxidation Changes at the Different IAPs

**Figure 2:**
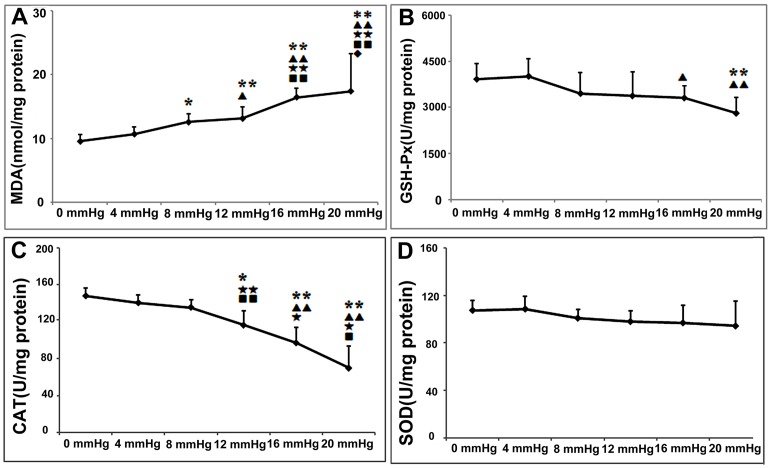
The effects of the different grades of nitrogen pneumoperitoneum on colonic pro-oxidant-antioxidant balance. A: Malondialdehyde (MDA), the product of lipid peroxidation. B–D: The antioxidant enzymes of glutathione peroxidase (GSH-Px), catalase (CAT), and superoxide dismutase (SOD). At 4 mmHg, the pro-oxidant-antioxidant balance was stable, and no significant differences in related parameters were detected between the 0- and 4-mmHg groups. Intra-abdominal pressures of ≥8 mmHg showed adverse effects on colonic pro-oxidant-antioxidant balance. Compared with that in the 0-mmHg group, the MDA concentrations in the 8, 12, 16, and 20-mmHg groups were significantly higher, whereas the CAT and GSH-Px concentrations tended to decline. No obvious changes in the SOD levels were detected. All data are shown as means ± SD. *P<0.05, **P<0.01, vs. 0 mmHg; ^▴^P<0.05, ^▴▴^P<0.01, vs. 4 mmHg; ^★^P<0.05, ^★★^P<0.01, vs. 8 mmHg. ^▪^P<0.05, ^▪▪^P<0.01, vs. 12 mmHg, ^⧫^P<0.05, vs. 16 mmHg.

As the IAP was increased, the pro-oxidant-anti-oxidant balance in the colon became increasingly disturbed. The levels of the lipid peroxidation product, MDA, increased significantly, whereas those of the antioxidant substances (namely, GSH-Px, CAT, and SOD) tended to decline (Figure 2). The differences in MDA and CAT levels between the 0-mmHg and IAH groups (12, 16, and 20 mmHg) were significant. We found that an IAP of 8 mmHg induced greater MDA production (8 mmHg vs. 0 mmHg: 12.55±1.28 vs. 9.54±1.10 nmol/mg protein, P = 0.017) even though the changes in other parameters were mild. These results indicate that an IAP of 8 mmHg for 90 min can cause increased oxidative damage to lipids, thus resulting in a pro-oxidant-antioxidant imbalance.

### Intestinal Permeability to FD-4at Different IAPs

**Figure 3:**
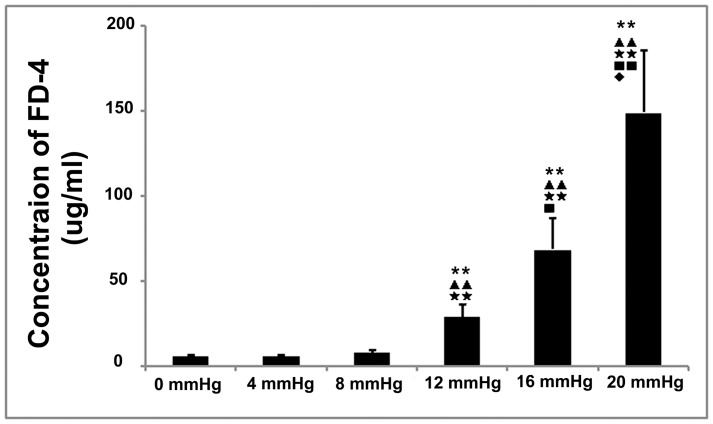
The effects of different grades of nitrogen pneumoperitoneum on intestinal permeability to FD-4. Intra-abdominal hypertension significantly stimulated the intestinal permeability to FD-4.Compared with that in the 0-mmHg group, the FD-4 concentration in the 12-, 16-, and 20-mmHg groups were increased 5.2-,12.4- and 26.7-fold, respectively. Slightly elevated intra-abdominal pressures of 4 and 8 mmHg did not affect the FD-4 concentrations. No significant differences were observed between the 0-mmHg group and the 4- and 8-mmHg groups. All data are shown as means ± SD. **P<0.01, vs. 0 mmHg; ^▴▴^P<0.01, vs. 4 mmHg; ^★★^P<0.01, vs. 8 mmHg; ^▪^P<0.05, vs. 12 mmHg; ^⧫^P<0.05, vs. 16 mmHg.

We determined the gut permeability to macromolecules using measurements ofFD-4 leakage from the gut cavity into the portal circulation. The FD-4concentrations in the portal blood of the IAH groups (12, 16, and 20 mmHg) were significantly higher than those in the non-IAH groups (0, 4, and 8 mmHg; Figure 3). In the non-IAH groups, although mild elevations in IAP did not induce prominent permeability damages, the portal blood concentrations of FD-4 tended to increase as the IAP increased.

### Morphological Damage at Different IAPs

**Figure 4:**
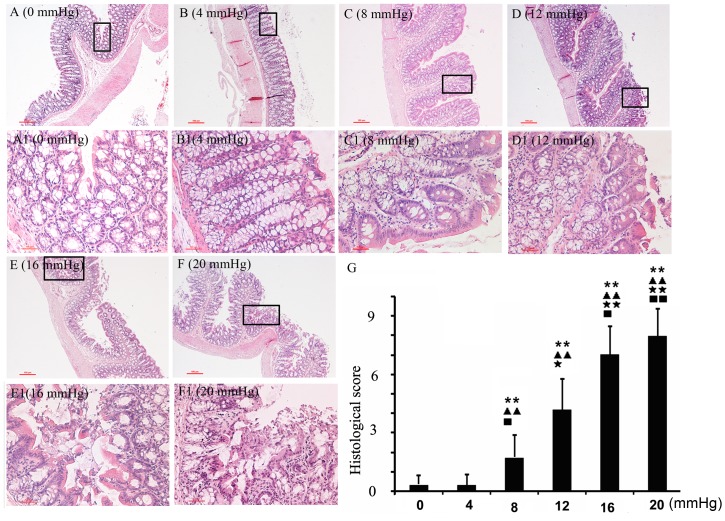
The effects of the different grades of nitrogen pneumoperitoneum on intestinal histologic damage. A–F (0–20 mmHg, ×100): Morphological changes under the different grades of nitrogen pneumoperitoneum. The parts in the black box are magnified and listed from A1 to F1 (0–20 mmHg, ×200). As the intra-abdominal pressure (IAP) increased, the severity of the histologic changes increased. At 8 mmHg, nitrogen pneumoperitoneum induced slight inflammatory cell infiltration. When the IAP reached 20 mmHg, almost no intact intestinal mucosa was observed. G: Histological scores in the different groups. Compared with the 0-mmHg group, the 8-, 12-, 16-, and 20-mmHg groups had significantly higher histological scores. All data are shown as means ± SD. **P<0.01, vs. 0 mmHg; ^▴▴^P<0.01, vs. 4 mmHg; ^★^P<0.05, ^★★^P<0.01, vs. 8 mmHg; ^▪^P<0.05, ^▪▪^P<0.01, vs. 12 mmHg.

H&E-stained sections and their corresponding histological scores, at the different IAPs, are shown in Figure 4; morphological damage, caused by IAH, is evident. IAPs of 12 mmHg or higher disturbed the epithelial integrity. The epithelial lining was destroyed and separated from the mucosa, which caused exposure and digestion of the lamina propria. Slightly elevated IAPs did not cause obvious damage to the mucosa, and the epithelial line remained intact. However, inflammatory cell infiltration was detected in the 8-mmHg group, and the histological damage score was significantly higher than that in the 4-mmHg and control groups.

### Expressions of Transmembrane Proteins at Different IAPs

**Figure 5:**
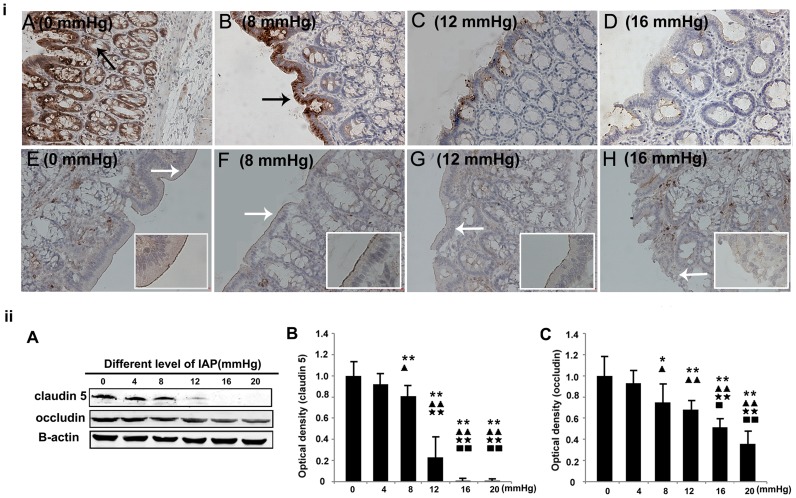
The effects of different grades of nitrogen pneumoperitoneum on the expression levels of TJ proteins. i: Immunohistochemical localization of claudin 5 and occludin (×200). Increased intra-abdominal pressures tended to reduce the expression levels of claudin 5 and occludin. A–D: Claudin 5. The black arrow indicates the positive signals for claudin 5 in the lateral membrane of the epithelia. E–H: Occludin. The white arrows indicate the positive signals for occludin in the apical cell borders of the colonic epithelia. ii: Western blotting results for claudin 5 and occludin. The expression levels of the proteins were normalized relative to actin. Compared with the 0-mmHg group, the 8-, 12-, 16-, and 20-mmHg groups had significantly reduced densities of claudin 5 and occludin. All data are shown as means ± SD. *P<0.05, **P<0.01, vs. 0 mmHg; ^▴^P<0.05, ^▴▴^P<0.01, vs. 4 mmHg;^★★^P<0.01, vs. 8 mmHg; ^▪^P<0.05, ^▪▪^P<0.01, vs. 12 mmHg.

To study the molecular mechanism of intestinal barrier dysfunction, we performed immunohistochemical staining and Western blotting examinationsforclaudin5 and occludin, which are the major types of TJs responsible for TJ permeability and paracellular transport [34]. As shown in Figure 5, claudin 5 was found in the lateral membrane of the intestinal epithelium and in the apical end of the colonic crypt. However, occludin was expressed mainly in the apical cell borders of the colonic epithelia. The expression levels of these proteins decreased as the IAP to which each group of animals was exposed increased. Compared with the control group, IAPs≥12 mmHg significantly lowered the expression of claudin 5 and occludin. In addition, even a smaller increase of the IAP, to 8 mmHg, also inhibited the expression of these proteins.

## Discussion

Enterogenic infection is an important factor in the development of sepsis and subsequent multiorgan failure (MOF) [35]–[38]. In the 1990s, Deitch et al. first proposed the concept that “the activation of intestinal mucosal immune system induced by bacterial translocation and permeability changes may be the motor of MOF” [39]. This opinion is also confirmed by results observed in the development of IAH [7], [11], [12], [40]–[44]. IAH, a common complication of critical illnesses, may be either a cause or a consequence of gastrointestinal (GI) dysfunction. In 2013, the difference between IAH in adults and children was first proposed. The IAP threshold in children, defined as 10 mmHg [1], is lower than that in adults. The adverse effects of IAH on GI mucosal damage is widely acknowledged [7], [11], [12], [40]–[44]. Nevertheless, the effects of slightly increased IAPs on the intestinal mucosa, and the related mechanism of action, remain unknown. Considering the unexplainable complication of abdominal discomfort in individuals who have experienced IAPs lower than the diagnostic standard, the intestinal effects of slightly elevated IAPs need to be investigated. The differences between our research and the existing studies on the effects of elevated IAPs on intestinal permeability and oxidative stress are shown in Table 3.

**Table 3:** Literature overview of the currently published animal experiments related to the present study.

The relevant point	Author (year)	Model	IAPs-duration	Relative detected index
**IAH on oxidant balances**	Eleftheriadis et al. [7] 1996	Rats,Pneumoperitoneum	15 mmHg-60 min	Mean arterial pressure, MDA level in gut mucosa, and bacterial translocation
	Gong et al. [6] 2009	Rats,Nitrogen pneumoperitoneum	20 mmHg-240 min	MPO, GSH, and GSH-Px levels in the ileum
	Kotidis et al. [5] 2012	RabbitsInflation of an intra-abdominal bag	12 mmHg-8 weeks	GSH, GSH-Px, and SOD levels in the rectus and abdominis muscles
**IAH on intestinal permeability and tight junction**	Gong et al. [42] 2009	Rats,Nitrogen pneumoperitoneum	20 mmHg-60, 120, and 240 min	Ultrastructural analysis of the tight junctions of the ileum
	Cheng et al. [12] 2013	Rabbits,Nitrogen pneumoperitoneum	15 and 25 mmHg-120, 240, and 360 min	Microcirculatory blood flow, intestinal permeability (FITC), and ultrastructural analysis of the tight junctions of the jejunum
**Slightly increased IAPs on intestinal function**	Cheng et al. [43] 2003	Rabbits,Nitrogen pneumoperitoneum	**10**, 20, and 30 mmHg-60, 120, and 240 min	Intestinal permeability (FITC and HRP-II)
	Yagci et al. [44] 2005	RabbitsInflation of an intra-abdominal bag	**10**, 15, 20, and 25 mmHg-720 min	Bacterial translocation
	Mogilner et al. [20] 2008	Rats,Air pneumoperitoneum	**3 and 6 mmHg**-120 min	Portal vein flow, liver damage, and hepatocyte apoptosis

MDA, malondialdehyde; GSH, glutathione; GSH-Px, glutathione peroxidase; MPO, myeloperoxidase; SOD, superoxide dismutase, FITC, fluorescein isothiocyanate.

In the present study, we experimentally demonstrated that elevated IAPs result in disturbances to histologic integrity, FD-4 permeability (Figure 3), TJ protein expression (Figure 5), and colonic oxidant balances in rats undergoing nitrogen pneumoperitoneum (Figure 2). The degree of damage was related to the degree of induced IAP. Slightly elevated IAPs showed similar undesirable effects, although to a lesser degree, compared with higher IAPs (equal to or higher than the threshold for IAH). In the 8-mmHg group, mild microscopic inflammatory infiltration and increased levels of MDA, a lipid peroxidation product, were detected. Although no significant differences in FD-4 permeability was found between the control animals and those exposed to an 8-mmHg IAP, TJ protein expression was markedly inhibited in the 8-mmHg group. These results demonstrate that a 90-min exposure to slightly elevated IAPs triggered intestinal injuries, without any detectable pathological or functional changes.

Creating an animal model to represent human intestinal pathology after exposure to slightly elevated IAPs is difficult, and the extent of IAP, the method of insufflation, and the animal model used need further discussion. In the present study, we chose rats exposed to nitrogen pneumoperitoneum, with IAPs of 0 (control), 4, 8, 12, 16, and 20 mmHg. The nitrogen pneumoperitoneum model used in this study is not a new model, and has been used in other studies [6], [45]. Compared with carbon dioxide or other gases, nitrogen is advantageous in that it avoids the absorption of foreign materials and the development of consequent pathophysiological changes, such as acidosis and excessive fluid absorption. As Blobner et al. reported, the peritoneum is able to absorb intra-abdominal carbon dioxide at IAP-levels up to 15 mmHg, which is closely related with the high solubility of carbon dioxide [46]. Conversely, nitrogen is almost insoluble, resulting in minimal adverse impacts (such as vasodilation) that can be ignored. Moreover, according to Schachtrupp et al., rat models may be more comparable to the conditions noted in children [47], where the standard threshold for IAH is 10 mmHg [1]. Hence, we included IAPs of 4 and 8 mmHg in the present study.

The IAH-induced reduction in blood flow in the superior mesenteric artery and intestinal mucosa is the direct cause of intestinal ischemia and hypoxia, which may also cause further mucosal injury [48], [49].Mesenteric ischemia, per se, regardless of IAP, is known to lead to decreased in histologic integrity, increased permeability, and increased bacterial translocation [50]–[52]. If the low-perfusion state continues, the systemic circulation and perfusion to other organs are further disturbed; moreover, BP and MAP are decreased [20], and cardiac output is diminished [53]. Similarly, we observed that as the degree of IAP increased, SBP, MAP, and APP were prominently decreased in rats with nitrogen pneumoperitoneum (Figure 1). The changes in these hemodynamic parameters were proportional to the histological and functional colon damage. As shown in Table 3, in addition to our group, only Mogilner JG et al. systematically investigated the adverse effects of slightly elevated IAPs in animals [20]. They found that an increased IAP, up to 3 mmHg, resulted in a significant decrease in portal vein and superior mesenteric artery blood flow and induced a reduction in MAP. When the IAP was increased to 6 mmHg, these changes were doubled. Although only a few liver histological changes were detected, hepatocyte proliferation and apoptosis occurred, thus supporting the presence of a mechanism of recovery from microdamage. The results of the present study were similar to those of Mogilner et al.We found that IAPs of 4 and 8 mmHgresulted in decreased SBPs and APPs (Figure 1), and maybe the cause of oxidant balance disturbance and enhanced permeability in the intestine.

Oxidative stress has been proposed as a common underlying mechanism for many factors that induce intestinal barrier dysfunction. In the development of IAH, oxidative stress is also important and may precede permeability enhancement [5]–[7]. As shown in Table 3, three groups have published related studies. Eleftheriadis et al. [7] reported that with an IAP elevation to 15 mmHg for 60 min, the jejunal mucosa microcirculation of rats was disturbed, followed by enhanced oxidant responses and bacterial translocation. The MDA levels also increased in the gut mucosa, liver, spleen, and lungs, and bacterial translocation toward the mesenteric lymph nodes, spleen, and liver increased. Gong et al. [6] further showed that IAH not only stimulated the process of oxidative stress, but also inhibited the anti-oxidation process. An IAP of 20 mmHg resulted in decreases in the glutathione and GSH-Px levels in the ileum of rats, consistent with our results. Moreover, the present study is the first to present supplementary data regarding the adverse effects of slightly increased IAPs. As shown in Figure 2, in the IAH rats, typical oxidation changes such as increased MDA levels and decreased CAT and GSH-Px levels were detected. An interesting finding is that the slightly increased IAP (8 mmHg) also affected the oxidant balance.

The intestinal mucosal barrier is the first line of GI defense. Gong et al. and Cheng et al. directly analyzed the influence of IAH on intestinal permeability [12], [42]. They found that IAH (20 mmHg for 60 min in rats or 15 mm Hg for 90 min in rabbits) led to significant increases in serum FD-4 levels. These permeability changes may be related to altered TJ protein levels. The results of the present study on rats with IAPs higher than 10 mmHg were consistent with their findings (Figures 3 and 5). In contrast, in the present study, we observed that slightly increased IAPs (4 mmHg for 90 min and 8 mmHg for 90 min) also resulted in the downregulation of TJ protein expression (Figure 5), although no significant changes in permeability (FD-4) were detected. In addition, the increased IAPs affected not only the expression levels of these proteins, but also the distribution of claudin 5. As shown in Figure 5, when the IAP was increased to 8 mmHg, the positive signals were mainly distributed in the epithelium, whereas the specific staining of claudin 5 in the colonic crypt cells was markedly reduced. Based on the results indicating oxidative damage and intestinal injury, we concluded that transient IAP elevations to 8 mmHg resulted in reduced TJ protein levels and, subsequently, increased permeability. These phenomena may be related to the pro-oxidant-antioxidant imbalance (Figures 2 and 5).

In conclusion, the present study supplemented the existing knowledge on the effects of elevated IAPs on the intestinal mucosa. In particular, not only IAH but also transient exposure to slightly elevated IAPs (e.g., 8 mmHg) led to mild ischemia and subsequent, undesirable disturbances in colonic permeability and pro-oxidant-antioxidant balance. Accordingly, in clinical practice, IAP elevations should be corrected as soon as possible to prevent the development of GI mucosal injury and the subsequent morbid cycle of sepsis that may ensue.
