# Correction: Effect of Acute, Slightly Increased Intra-Abdominal Pressure on Intestinal Permeability and Oxidative Stress in a Rat Model

**DOI:** 10.1371/journal.pone.0115133

**Published:** 2014-12-03

**Authors:** The PLOS ONE Staff

The following information is missing from the Funding section: The work was supported by The National Natural Science Foundation of China 81441061. Please refer to the complete funding statement here.

The work was supported by the following: Peking University - Tsinghua University Joint Center for Life Sciences, Beijing science and technology plan - Z141107002514020, and The National Natural Science Foundation of China 81441061. The funders had no role in study design, data collection and analysis, decision to publish, or preparation of the manuscript.
